# Cyclodextrins inhibit TRPV1 and TRPA1 activation-induced nociception via cholesterol depletion

**DOI:** 10.1016/j.jlr.2025.100844

**Published:** 2025-06-16

**Authors:** Andrea Nehr-Majoros, Lajos Karakai, Maja Payrits, Noémi Bencze, Ágnes Kemény, György Sétáló, Rita Börzsei, Csaba Hetényi, Zsuzsanna Helyes, Éva Szőke

**Affiliations:** 1 Department of Pharmacology and Pharmacotherapy & Centre for Neuroscience, Faculty of Medicine, University of Pécs, Pécs, Hungary; 2 National Laboratory for Drug Research and Development, Budapest, Hungary; 3 Department of Physiology and Biochemistry, University of Veterinary Medicine, Budapest, Hungary; 4 Department of Medical Biology, Faculty of Medicine, University of Pécs, Pécs, Hungary; 5 Hungarian Research Network, Chronic Pain Research Group, University of Pécs (HUN-REN PTE), Pécs, Hungary

**Keywords:** analgesia, neurogenic, inflammation, cholesterol, formalin, mustard oil, resiniferatoxin

## Abstract

The nociceptive Transient Receptor Potential Vanilloid 1 (TRPV1) and Ankyrin 1 (TRPA1) channels are predominantly expressed on peptidergic sensory nerves, being involved in pain sensation and neurogenic inflammation induced by local release of pro-inflammatory neuropeptides in the innervation area. Their activation is facilitated by cholesterol-rich lipid microdomains (lipid rafts) in the plasma membrane. Cyclodextrin (CD) derivatives deplete cholesterol from membrane rafts, reducing receptor activation in vitro, anticipating in vivo analgesic effects. We compared three different CD derivatives selected based on our previous results: random methylated β-cyclodextrin, (2-hydroxypropyl)-β-cyclodextrin and sulfobutylether-β-cyclodextrin. The effects of the topical CD pretreatments were evaluated in acute pain and neurogenic vasodilatation models in mice 30 min after TRPV1 (resiniferatoxin) or TRPA1 (formalin or mustard oil) receptor agonist administration. Intraplantar CD pretreatments significantly reduced the duration of nocifensive behaviors during the neurogenic inflammatory phase of the formalin test, as well as mechanical, but not thermal hyperalgesia following resiniferatoxin injection. CD-pretreatment significantly reduced mustard oil-induced acute neurogenic vasodilatation in the mouse ear and decreased the total cholesterol content in the plantar skin and ear tissues. Cholesterol depletion was restored by cholesterol loaded CDs. However, overloading cells with cholesterol did not significantly affect cholesterol depletion. In silico modeling showed that the methylated derivative RAMEB has different cholesterol binding mode compared to HPBCD and SBECD. We present the first in vivo results showing that these CD derivatives are promising agents for exerting peripheral analgesia and anti-inflammation via cholesterol depletion, also supported by our in vitro and in silico findings.

The nonselective cation channels Transient Receptor Potential Vanilloid 1 and Ankyrin 1 (TRPV1 and TRPA1, respectively) are continuously emerging targets for pain (1, 2, 3, 4, 5). These nociceptive cation channels are often co-expressed on capsaicin (CAPS)-sensitive peptidergic sensory nerves (6, 7, 8). Nociceptive C-fibers transmit the pain signal to the central nervous system, while inflammatory neuropeptides, such as calcitonin gene-related peptide (CGRP) and Substance P (SP), are locally released from them, resulting in vasodilation and plasma protein extravasation in the innervated area, called neurogenic inflammation (7, 9, 10). These ion channels show a polymodal activation profile responding to a variety of endogenous and exogenous stimuli. TRPV1 is the primary target of some vanilloid-type, pungent compounds (CAPS, resiniferatoxin (RTX), N-oleoyldopamine), lipids (e.g anandamide, N-arachidonoyl-dopamine, various lipoxygenase products), heat exceeding 43°C or protons (pH < 6) (11, 12, 13, 14, 15, 16, 17, 18, 19, 20, 21). TRPA1 activation is induced by noxious cold (T<17°C), electrophilic isothiocyanates from plants (e.g allyl-isothiocyanate (AITC) from mustard oil (MO), cinnamaldehyde), thiosulfates (e.g diallyl sulfide or diallyl disulfide), other irritants (e.g formaldehyde), cannabinoids (e.g tetrahydrocannabinol), and mechanical stimuli (21, 22, 23, 24, 25, 26, 27). These receptors are highly druggable, and great efforts have been put into antagonist development in the last decades. Although drug candidates were effective, they failed in clinical trials due to severe, even life-threatening side effects such as hyperthermia and increased thermonociceptive threshold, potentially leading to burn injuries (4, 5, 28). Thus, novel approaches are required to inhibit the activation of these receptors, which differ from classical antagonism.

The lipid raft model suggests that lateral organization of the plasma membrane by lipid-lipid interactions leads to the disposition of receptors to the more ordered raft regions, thus facilitating receptor activation (29). These nanomolar-scale (10–200 nm) microdomains are extremely rich in special lipid constituents such as cholesterol, sphingolipids, or gangliosides. It is proven that lipid raft disruption highly affects the function of raft-residing receptors (30, 31, 32). Several possibilities exist to disrupt plasma membrane integrity: depletion or sequestration of lipid constituents (e.g cholesterol), hydrolysis or inhibition of their synthesis (e.g sphingolipids, gangliosides) (33, 34). Beta-cyclodextrins (BCDs) efficiently sequester cholesterol and deplete it from the plasma membrane (35, 36, 37). These cone-shaped cyclic oligosaccharides are composed of 7 glucopyranose units, forming a ring with a hydrophilic outer surface and a hydrophobic inner cavity. This structure makes them appropriate to form host-guest inclusion complexes with poorly water-soluble molecules, such as cholesterol (38, 39). With their given cavity diameter of 0.60–0.65 mm and height of 0.78 mm, BCDs form a 2:1 BCD: cholesterol complex (40, 41). Besides the native form, several substituted BCDs exist, which, depending on the characteristics of their substituents, possess different chemical properties. The substituents also affect the complexation affinity of the distinct BCD derivatives (42, 43, 44).

Given the fact that functional TRPV1 and TRPA1 are mostly residing in the lipid raft regions, extensive research is conducted to reveal the potential inhibitory mechanisms of TRP ion channels via lipid raft disruption (45, 46, 47). Several research groups proved that lipid raft disruption inhibited TRP receptor activation in vitro in different neuronal and cell line models. Lipid raft disruption negatively alters TRPV1 (48, 49, 50, 51, 52, 53, 54, 55, 56) and TRPA1 (57, 58, 59, 60). Our research group has demonstrated earlier that pretreatment of mice with different lipid raft disruptors exerted analgesic effect on TRPV1- and TRPA1-mediated acute pain responses in mice (61, 62). Others showed that the lipid raft disruptor methyl-β-cyclodextrin (MCD) exerted antinociceptive effect on RTX-induced neuropathic hyperalgesia in mice (63). Considering the relatively high cytotoxicity of MCD, we previously screened 8 chemically distinct CD derivatives in respect of their safety, structural effects on the plasma membrane via cholesterol depletion and functional effects on TRPV1 and TRPA1 ion channels (51). The concentrations in which these derivatives exerted significant inhibiting effect but were well tolerable for cells were defined.

Here we tested the effects of three promising, structurally different CD derivatives, randomly methylated beta-cyclodextrin (RAMEB, methylated and neutral), (2-hydroxypropyl)-beta-cyclodextrin (HPBCD, non-methylated and neutral), and sulfobutylether-beta-cyclodextrin sodium salt (SBECD, anionic), on TRPV1- and TRPA1-induced inflammatory and pain responses in mice. These CD derivatives have preferential toxicity profiles in the applied concentrations but have favorable cholesterol complexing ability (51). These three derivatives are already widely used as active compounds and/or as excipients in the pharmaceutics; however, their analgesic and anti-inflammatory potential has not yet been investigated in detail (64, 65, 66, 67).

## Materials and Methods

### Animals and ethics

In all experiments, 12- to 16-week-old male NMRI mice were used. Animals originated from and were kept in the Laboratory Animal House of the Department of Pharmacology and Pharmacotherapy of the University of Pécs at 24–25°C, under a 12–12 h light–dark cycle, and were provided with standard rodent chow and water ad libitum. All experimental procedures were carried out according to the 1998/XXVIII Act of the Hungarian Parliament on Animal Protection and Consideration Decree of Scientific Procedures of Animal Experiments (243/1988). The study was approved by the Ethics Committee on Animal Research of Pécs University according to the Ethical Codex of Animal Experiments, and a license was obtained (license No.: BA02/2000-22/2023). The minimal required sample size was calculated with a priori analysis. The effect of CD derivatives compared to vehicle controls was investigated by pretreating the animals 30 min before the ion channel activator, inflammatory compounds were applied.

### Formalin-induced acute somatic nocifensive behavior test

30 min before the i.pl. administration of the TRPA1 agonist formalin (20 μl, 2.5%) into the right hind paw of the animals, CD pretreatment was applied (20 μl CD solution dissolved in physiological saline, i.pl.). Formalin injection immediately evoked nocifensive reactions (shaking, licking, lifting, and holding of the affected hind paw), the duration of which was recorded between 0–5 min (first phase) and 20–45 min (second phase) (61, 62, 68, 69). The nocifensive reaction of the first phase is arising from the direct activation of sensory nerve terminals, while in the second phase, neurogenic inflammatory mechanisms occur due to the release of acute inflammatory mediators (70).

### Resiniferatoxin-induced acute thermal allodynia and mechanical hyperalgesia model

The ultrapotent TRPV1 agonist RTX (20 μl, 0.1 μg/ml) was injected i.pl. into the right hind paw of the animals. RTX injection is known to induce thermal allodynia and mechanical hyperalgesia via acute neurogenic inflammation, involving both peripheral and central mechanisms (71, 72). On days −2 and −1 before RTX injection control thermo- and mechanonociceptive threshold measurements were performed by increasing the temperature hot plate (IITC Life Science) and by the dynamic plantar aesthesiometer (DPA), respectively. Measured control values were taken as self-control. On the day of the experiment, CD pretreatment (20 μl, i.pl.) was applied 30 min before RTX administration. Thermonociceptive threshold values were registered 10, 20, and 30 min, while mechanical hyperalgesia was investigated 30 and 90 min after RTX administration, as described earlier (73, 74, 75) Vehicle pretreatment with RTX treatment was taken as a positive control.

### Mustard oil-induced acute neurogenic vasodilation in the ear

To detect the effect of CD pretreatment on vascular changes during the initial period of acute inflammation in vivo, the TRPA1 agonist MO-induced acute skin inflammation model was applied, with minor changes compared to as described previously (76, 77). Anesthesia was induced by ketamine (100 mg/kg) with xylazine (5 mg/kg) and was repeated in half - one-thirdtoto doses during the experiment if necessary. Both ears were pretreated with ethanol (control group) or CDs dissolved in ethanol in the appropriate concentration by smearing (10-10 μl on both sides of the ears). 30 min later, the left ear was smeared with 1% MO dissolved in paraffin oil (10-10 μl on both sides). The right ear served as a reference, paraffin oil (10-10 μl on both sides) was applied to it. Immediately after the MO application, PeriCam Laser Speckle Contrast Analysis (PeriCam PSI System) was performed for 20 min to monitor cutaneous blood perfusion alterations in the mouse ear. To ensure simultaneous scanning of both ears, an area of 5.9 × 4.0 cm was scanned. Measuring distance was set between 16.8 and 17.3 cm, and the resolution to 0.16 mm. The frame rate was 44 images/s. Two regions of interest (ROIs) were assigned, representing the total area of both ears. The mean perfusion of the left ear (treated) was compared to that of the right ear (reference), and the percentage difference values of the two ears were compared between the groups. Mouse ear thickness was measured with an engineer’s micrometer before (baseline) and 60, 120, 180, and 240 min after the MO treatment to detect oedema formation. Post-treatment measured values were compared to baseline values. During the whole experiment, mice were placed on a heating pad to maintain core body temperature.

### Colorimetric cholesterol content measurement

To measure the cholesterol level in the mouse ear or the plantar skin after CD pretreatment ab65359 Abcam Cholesterol/Cholesteryl Ester Quantitation Assay kit was used, according to manufacturer’s protocol. Briefly, mice were pretreated with saline (control group) or CD solutions by smearing (10-10 μl on both sides of the ears) or by i.pl. injection (20 μl) for 30 min. Following incubation mice were sacrificed, and ear or plantar skin tissues were harvested. Tissue samples from 3-3 mice from the same groups were pooled and handled as one sample to achieve detectable cholesterol levels. After washing with cold phosphate-buffered saline (PBS) lipids were extracted by resuspending the samples in 200 μl Chloroform: Isopropanol:NP-40 (7:11:0.1) mixture/10 g tissue in a micro-homogenizer (20,000 rpm, 2 × 30 s) on ice. Homogenates were centrifuged (10 min, 15,000 *g*, 4°C) and the supernatant (organic phase) was collected and air dried (50°C). Lipid extract was resuspended in 200 μl Assay Buffer and the assay was performed by adding the appropriate reagents as specified by the manufacturer. After incubation on an orbital shaker (37°C, 60 min) optical density at 570 nm (OD570) was recorded on a microplate reader (CLARIOstar Plus, BMG LABTECH) and recordings were corrected with sample weight and dilution factors. Cholesterol concentration was determined by comparing the absorbance values to a standard curve generated from known concentrations of cholesterol standards.

### Filipin III fluorescent staining of cholesterol

Filipin III is a bacteria-originated macrolide antibiotic frequently used as a fluorescent dye for visualizing cholesterol intracellularly (78, 79). Native CHO cells were seeded on 10-well CELLview™ cell culture slides at a density of 5,000 cells/100 μl complete Dulbecco’s Modified Eagle’s Medium (DMEM) per well, and incubated overnight (37°C, 5% CO_2_). CD derivatives were dissolved in extracellular solution (ECS) in appropriate concentrations. Following PBS washing cells were incubated in 100 μl of ECS (control) or 100 μl of CD solution dissolved in ECS for 30 min (37°C, 5% CO_2_). In the restorative approach cells were incubated with cholesterol-loaded CD (CLC) solution in complete DMEM for 30 min (37°C, 5% CO_2_). In another group, cells were overloaded with cholesterol prior to CD treatment by 30-min CLC treatment (37°C, 5% CO_2_). In the control group only CD treatment was applied. After PBS washing cells were fixed with 4% paraformaldehyde (PFA, 100 μl/well) for 30 min, and incubated with 100 μl of glycine in PBS (1,5 mg/ml) at room temperature to quench the PFA. Cholesterol was stained with 100 μl of Filipin III solution (0.05 mg Filipin III/ml PBS/10% fetal bovine serum (FBS)) for 2 h at room temperature. Cells were washed three times with PBS after every step. Finally, cells were covered with ProLong™ Glass Antifade Mountant (Invitrogen). Staining was visualized using an Olympus Fluoview-1000 system. Micrographs were generated using an Olympus Fluoview-1000 system on an Olympus IX81 microscope stage equipped with an Olympus DP70 digital camera and through an Olympus UPlan FL N, Phase2 objective (40×/0.75), with mercury lamp DAPI filters (excitation: 335–375 nm, emission: 440–500 nm). Image size was set at 680×512 and ISO at 200. Mean pixel intensity per cell was measured in ImageJ software. Background correction was performed by subtracting the mean intensity of the non-cell-containing background of the same sized area from the mean intensity value of each cell. Mean intensity values were compared between the corresponding groups (minimum 100 cells from 3 independent experiments/group).

### Preparation of the host (CD) and guest (cholesterol) molecules

BCD was obtained from its crystal structure with cyclodextrin glycosyltransferase (PDB code: 3cgt (80)). Substituents of all CD derivatives were added in PyMol (The PyMOL Molecular Graphics System, Version 2.5.0 Schrödinger, LLC.). The number and positions of substituents were determined based on the degree of substitutions (DS) as well as literature data. According to the DS numbers provided by the manufacturer (4.5, 6.3, and 12), 5, 6, and 12 substituents were attached to HPBCD, SBECD, and RAMEB, respectively. For HPBCD, two, one, and two 2-hydroxy-propyl substituents were added to C2, C3, and C6 positions, respectively. For SBECD, the positions of the sulfobutylether substituents are the same as indicated in a previous paper (81). For RAMEB, four, one, and seven methyl groups were connected to C2, C3, and C6 positions. Cholesterol was built in Maestro (Schrödinger Release 2024-2: Maestro, Schrödinger, LLC, 2024., n.d.). The raw structures were energy-minimized by the semiempirical quantum chemistry program package, MOPAC (MOPAC2016, James J. P. Stewart, Stewart Computational Chemistry, http://OpenMOPAC.Net (2016)., n.d.) with PM7 parametrization. The gradient norm was set to 0.001. Force calculations with positive force constant matrices were applied to the energy-minimized structure. These optimized structures were used for docking calculations.

### Docking calculations

Docking calculations were performed by AutoDock 4.2.6. (82) using minimized and equilibrated cholesterol and the host structures. Gasteiger–Marsilli partial charges were assigned to both the guest and host atoms in AutoDock Tools (82), and a united atom representation was applied for nonpolar moieties. Flexibility was allowed at all active torsions of the guests, and the hosts were treated rigidly. The grid box was centered on the middle of host molecules and was set to be at least 3 times larger than the longest dimension of the hosts, including 120x120x120 grid points at a 0.375 Å spacing by AutoGrid 4. One hundred blind docking runs were performed in each calculation. The Lamarckian genetic algorithm and the pseudo-Solis and Wets local search with a maximum number of 300 iterations and 25 million energy evaluations, and 150 population size were applied as in (83). The generated 100-ligand-binding modes were clustered and ranked based on their calculated free energy of binding values and structural similarity with a Root Mean Squared Deviation (RMSD) cutoff of 3.5 Å. The Rank 1 was chosen as the representative cholesterol structure, and its calculated free energy of binding (ΔG_b_) was also calculated.

### Materials

The following CD derivatives were offered by CycloLab Cyclodextrin Research and Development Ltd. (Budapest, Hungary): Randomly methylated beta-cyclodextrin (RAMEB, CY-2004.1), (2-Hydroxypropyl)-beta-cyclodextrin (HPBCD, CY-2005.2) and Sulfobutylether-beta-cyclodextrin sodium salt (SBECD, CY-2041.2). CDs were dissolved in physiological saline (NaCl 0.9%, B Braun Hungary) or in ethanol (Scharlab Hungary Ltd., Hungary, PubChem CID: 702) and in ECS for the in vivo and the *in* vitro expreiments, respectively, to reach final concentrations of 3 mM (RAMEB) or 10 mM (HPBCD, SBECD), based on previous in vitro investigations. RTX (Sigma-Aldrich, Inc., St. Louis, MO, USA; PubChem CID: 5702546) was dissolved in ethanol to yield a 1 mg/ml stock solution, and further diluted with saline to final concentration of 0.1 μg/ml. Formalin was diluted with phosphate-buffered saline (PBS, PubChem CID: 24978514) from a 6% formaldehyde stock solution (Molar Inc., Hungary, PubChem CID: 712). Mustard oil (MO, 100 mg/ml, PubChem CID: 5971) and paraffin oil (PubChem CID: 68245) were purchased from Merck KgaA, Germany. MO was used as a 100-times dilution (1%) in paraffin oil. Chloroform was purchased from Sigma-Aldrich, Inc. (PubChem CID: 6212), isopropanol from Chem-Lab Analytical (Belguim, PubChem CID: 3776) and NP-40 from Thermofisher Scientific (Waltham, MA, USA). Complete DMEM contained the following: 500 ml low glucose (1 g/L) DMEM (Capricorn Scientific GmbH, Germany) supplemented with 50 ml FBS (Euroclone, Italy), 5 ml GlutaMAXTM-I (100X) solution (Thermo Fisher Scientific, Waltham, MA, USA), 5 ml GibcoTM MEM non-essential amino acid solution (100X) and 500 μl penicillin-streptomycin mixture (Lonza Group). Filipin III dye was purchased from Sigma-Aldrich, Inc.

### Statistical analysis

A priori sample size calculation was performed by G∗Power (Department of Psychology at Heinrich Heine University Düsseldorf (HHU), Germany) for detecting a large effect size (0.40) with 95% power at an alpha level of 0.05. All the animal experiment data are presented as means ± standard error of mean (SEM) of 4–8 animals per group, depending on the model (see figure legends). Statistical analysis was performed using GraphPad Prism 8.0.1 (GraphPad) by repeated measures (RM) two-way analysis of variance (ANOVA) with the Geisser-Greenhouse correction or ordinary one-way ANOVA. ANOVA was followed by Dunnett’s or Sidak’s multiple comparison test. During the analysis of colorimetric cholesterol measurements Shapiro–Wilk normality testing and unpaired *t* test were performed. In cellular measurements, statistical analysis was performed from a minimum of three independent experiments. Data were evaluated using GraphPad Prism 8.0.1 (GraphPad, La Jolla, CA, USA) software. D’Agostino-Pearson normality testing, followed by the Kruskal–Wallis test and Dunn's multiple comparison test, were applied to compare control and treated groups. Microscopic images were evaluated using ImageJ 1.53 software.

## Results

### CD derivatives reduce TRPA1 activation-induced acute nocifensive behaviors

**Fig. 1:**
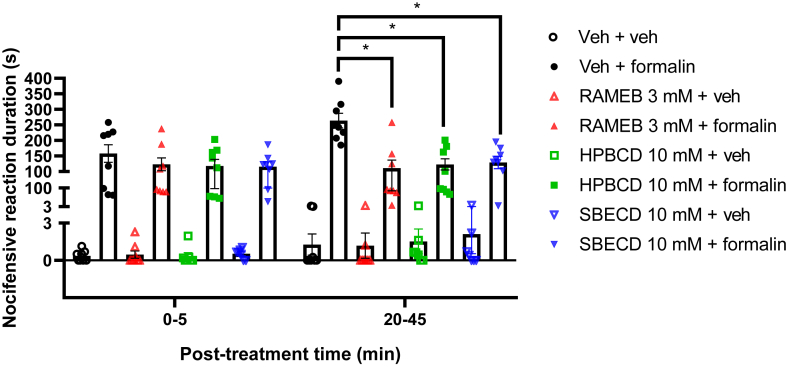
Effect of RAMEB (3 mM), HPBCD (10 mM) and SBECD (10 mM) on the duration of formalin-induced acute nocifensive behavior. Data are represented as means ± SEM of n = 7–8 animals/group. Statistical analysis was RM two-way ANOVA, followed by Tukey’s multiple comparison test (∗*P* < 0.0001 CD-pretreated groups vs. saline-pretreated control group).

The CD derivative-induced effect on TRPA1-mediated acute somatic nociception was investigated by recording the duration of nocifensive behavior following intraplantar (i.pl.) formalin treatment. In the saline-pretreated control group the animals spent 157.8 ± 80.11 s and 264.0 ± 23.63 s with licking, lifting, shaking, or holding the affected paw in the first and the second phases of the experiment, respectively. CD pretreatment did not significantly alter the duration of the nocifensive behavior derived from the direct chemical activation of TRPA1 receptors and sensory nerve terminals (first phase), while it was significantly decreased in the neurogenic inflammation-related second phase, with recorded values of 111.2 ± 25.7 s, 122.8 ± 17.94 s and 129.0 ± 20.16 s for 3 mM RAMEB, 10 mM HPBCD and 10 mM SBECD pretreatment, respectively (Fig. 1). Saline and CD pretreatment alone did not have any significant effect on the duration of the nocifensive behavior of mice.

### CD derivatives alleviate TRPV1 activation-induced acute mechanical, but not thermal hyperalgesia

**Fig. 2:**
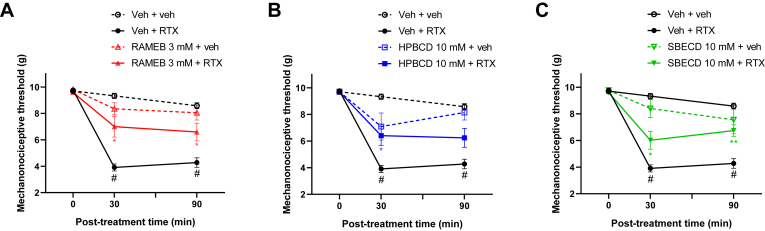
Effect of 3 mM RAMEB (A), 10 mM HPBCD (B) and 10 mM SBECD (C) on the RTX-induced mechanical hyperalgesia. Data are represented as means ± SEM of n = 4 animals/saline + saline control group, n = *6*/saline + RTX group and n = 7–8/CD-pretreated groups. Statistical analysis was RM two-way ANOVA with the Geisser-Greenhouse correction and Dunnett’s multiple comparison test (∗*P* < 0.05, ∗∗*P* < 0.005: CD-pretreated groups vs. saline + RTX group; #*P* < 0.0001: saline + saline group vs. saline + RTX group).

**Fig. 3:**
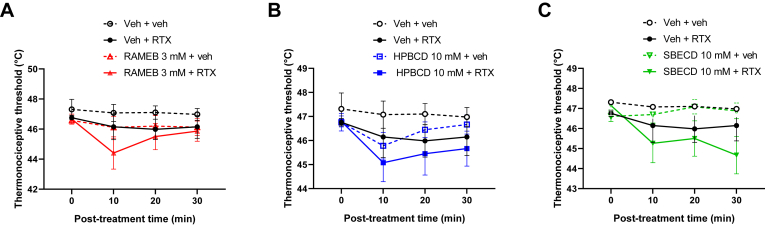
Effect of 3 mM RAMEB (A), 10 mM HPBCD (B) and 10 mM SBECD (C) on the RTX-induced thermal allodynia. Data are represented as means ± SEM of n = 4 animals/Veh + veh control group, n = 6/Veh + RTX group and n = 7–8/CD-pretreated groups. Statistical analysis was RM two-way ANOVA with Dunnett’s multiple comparison test (∗*P* < 0.05, ∗∗*P* < 0.005: CD-pretreated groups vs. saline + RTX group; #*P* < 0.0001: saline + saline group vs. saline + RTX group).

TRPV1 receptor activation by its agonist RTX resulted in reduced mechanonociceptive threshold of the animals: compared to the baseline values of 9.76 ± 0.14 g, after RTX treatment the mechanonociceptive threshold was reduced to 3.91 ± 0.27 g and 4.28 ± 0.36 g in the saline-pretreated group 30 min and 90 min following i.pl. RTX treatment, respectively. CD-pretreatment alleviated the acute mechanical hyperalgesia, resulting in the following mechanonociceptive threshold values: 7.0 ± 0.78 g, 6.40 ± 0.72 g, and 6.02 ± 0.67 g 30 min after RTX administration, 6.59 ± 0.67 g, 6.24 ± 0.72 g, and 6.74 ± 0.44 g 90 min after RTX administration for 3 mM RAMEB, 10 mM HPBCD, and 10 mM SBECD, respectively (Fig. 2). The RTX-induced thermal allodynia was not influenced by the CD pretreatment (Fig. 3).

### CD derivatives reduce TRPA1 activation-induced acute neurogenic vasodilation in the skin

**Fig. 4:**
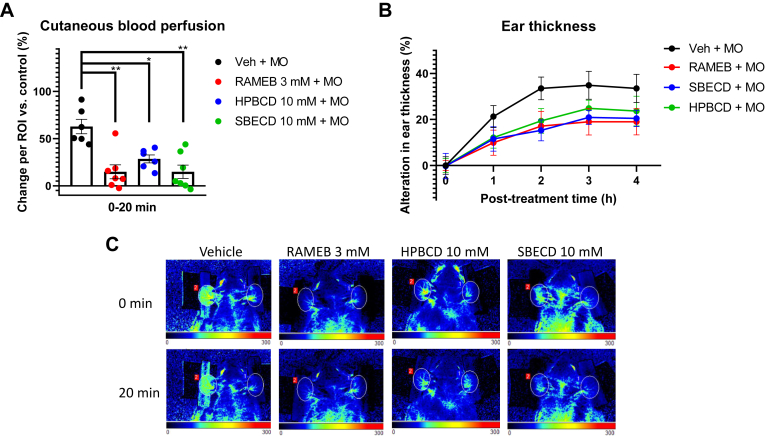
Effect of 3 mM RAMEB, 10 mM HPBCD and 10 mM SBECD on MO-evoked acute skin inflammation in mouse ear. A: Percentage change per region of interest (ROI) of the cutaneous blood flow of the MO-treated ear compared to the vehicle-treated control ear. Data is represented as means ± SEM of n = 6 animals/Veh + MO control group and n = 7–8/CD-pretreated groups. Ordinary one-way ANOVA with Dunnett’s multiple comparison test (∗*P* < 0.005, ∗∗*P* < 0.0001 vs. control). B: Alterations in ear thickness following MO treatment. Data is represented as means ± SEM of n = 6 animals/Veh + MO control group and n = 7–8/CD-pretreated groups. RM two-way ANOVA with the Geisser-Greenhouse correction and Sidak’s multiple comparison test. C: Representative images of the PeriCam laser speckle recordings of the MO-treated mouse ear. Applied pretreatments (vehicle (control) or CD) and the time of the recording are indicated on the image.

CD pretreatment was applied to mouse ear to detect its effects in acute skin inflammation induced by the TRPA1 receptor agonist MO. The right ear of the mice served as a self-control with only vehicle (paraffin oil) administration, and the blood perfusion values of this reference ear were compared to those of the MO-treated left ear. Laser speckle contrast analysis results showed that in the vehicle (ethanol)-pretreated control group, the cutaneous blood perfusion elevated in the affected ear of mice by 61.75 ± 6.52%, compared to the reference ear. However, in CD-pretreated groups, this rise was diminished to values of 7.336 ± 2.90%, 28.50 ± 4.24% and 14.88 ± 7.10% for 3 mM RAMEB, 10 mM HPBCD and 10 mM SBECD, respectively (Fig. 4A). Ear thickness measurements with engineers’ micrometer 1, 2, 3, and 4 h following MO treatment showed that due to the one-dose MO, smearing oedema formation occurred in the treated ear, which was slightly decreased by CD pretreatment, but the drop in the ear thickness values was not significant (Fig. 4B).

### CD treatment reduces the total cholesterol content of the plantar skin and ear

**Fig. 5:**
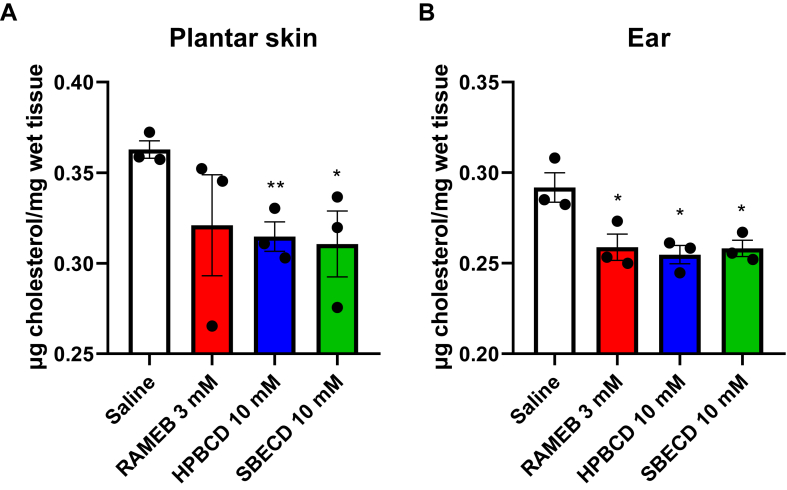
Total cholesterol content of mouse plantar skin (A) or mouse ear (B) tissue after 30-min CD treatment. Data is represented as means ± SEM (n = 3, unpaired *t* test following Shapiro-Wilk normality testing, ∗*P* < 0.05, ∗∗*P* < 0.01).

To observe the in vivo cholesterol depletion of CD derivatives following topical administration, the total cholesterol content of mouse plantar skin and ear tissues were measured 30 min following i.pl. injection or smearing on the ear surface, respectively. Colorimetric Abcam Cholesterol Assay kit results showed that compared to the saline-treated controls (0.363 ± 0.005 μg/mg) in mouse plantar skin the total cholesterol content was reduced to 0.321 ± 0.028 μg/mg, 0.315 ± 0.008 μg/mg, and 0.311 ± 0.018 μg/mg after 3 mM RAMEB, 10 mM HPBCD and 10 mM SBECD, respectively (Fig. 5A). After smearing the CD derivatives on the ear of the mice, the total cholesterol value of the saline-treated control was 0.292 ± 0.008 μg/mg, while in the CD-treated groups the results were the following: 0.259 ± 0.007 μg/mg, 0.255 ± 0.005 μg/mg and 0.258 ± 0.005 μg/mg after 3 mM RAMEB, 10 mM HPBCD and 10 mM SBECD, respectively (Fig. 5B), which results showed that topical CD administration significantly reduced cholesterol content in vivo.

### CD treatment can be reversed by cholesterol restoration, but cholesterol overload does not prevent depletion

**Fig. 6:**
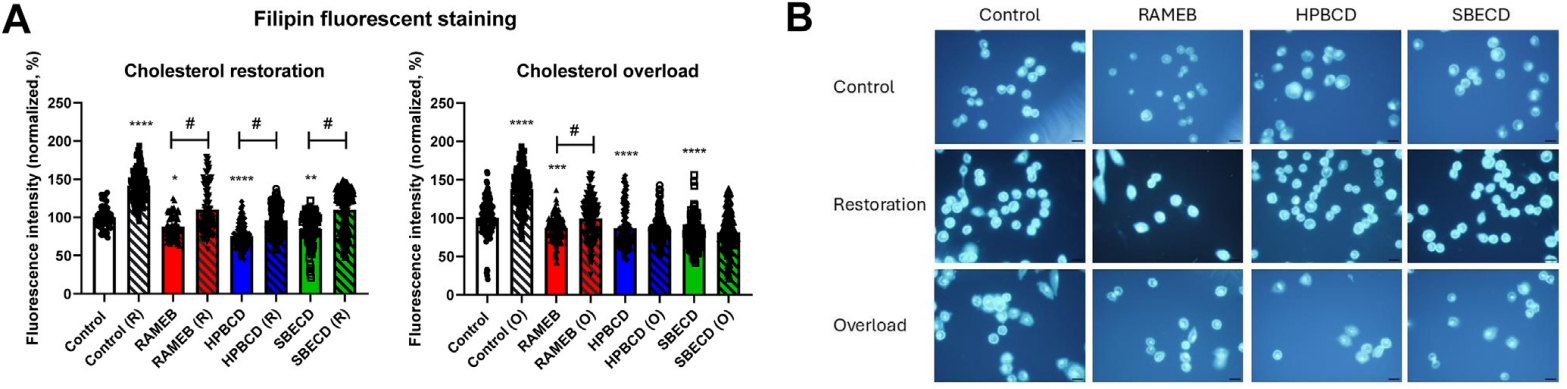
Effect of 30-min CD treatment on the cholesterol content of native CHO cells with and without cholesterol restoration (R) or cholesterol overload (O) applied after or before CD treatment, respectively. A: Quantification of fluorescent signals of the Filipin III dye. Normalized fluorescence intensity values are presented in percentage as means ± SEM of 150–200 CHO cells from 3 independent experiments (Kruskal-Wallis test, Dunn’s multiple comparison test; ∗*P* = 0.02, ∗∗*P* = 0.007, ∗∗∗*P* = 0.0007, ∗∗∗∗*P* < 0.0001 vs. control; #*P* < 0.008, ##*P* < 0.0001). B: Representative images after Filipin III staining of untreated (control) and CD-treated cells. Scale bar: 20 μm.

The cholesterol-depleting ability of CD derivatives and their dynamic nature were investigated by Filipin III staining by fluorescent microscopy. The effect of CDs on plasma membrane cholesterol levels was investigated in three groups with different approaches: (1) CD treatment, (2) cholesterol restoration following CD treatment, and (3) cholesterol overload prior to CD treatment. Accumulation of Filipin III staining was detected in both the plasma membrane and perinuclear compartments of control cells, indicating the presence of cholesterol (Fig. 6B). 3 mM RAMEB, 10 mM HPBCD, and 10 mM SBECD treatment resulted in a significant decrease in cellular cholesterol content. Restoring cholesterol by CLC resulted in recovery of the mean fluorescent intensity of the cells, resulting in 110.2 ± 2.7%, 95.8 ± 1.3% and 110.1 ± 2.1% for 3 mM RAMEB, 10 mM HPBCD and 10 mM SBECD, respectively (Fig. 6A). However, overloading the cells with cholesterol by CLC (overloaded untreated control: 138.0 ± 2.0%) only prevented the cells from cholesterol depletion by 3 mM RAMEB (99.5 ± 2.0%, but not 10 mM HPBCD and 10 mM SBECD (85.1 ± 1.2 and 81.3 ± 2.4%, respectively) (Fig. 6A).

### RAMEB, HPBCD, and SBECD have different cholesterol-binding characteristics

**Fig. 7:**
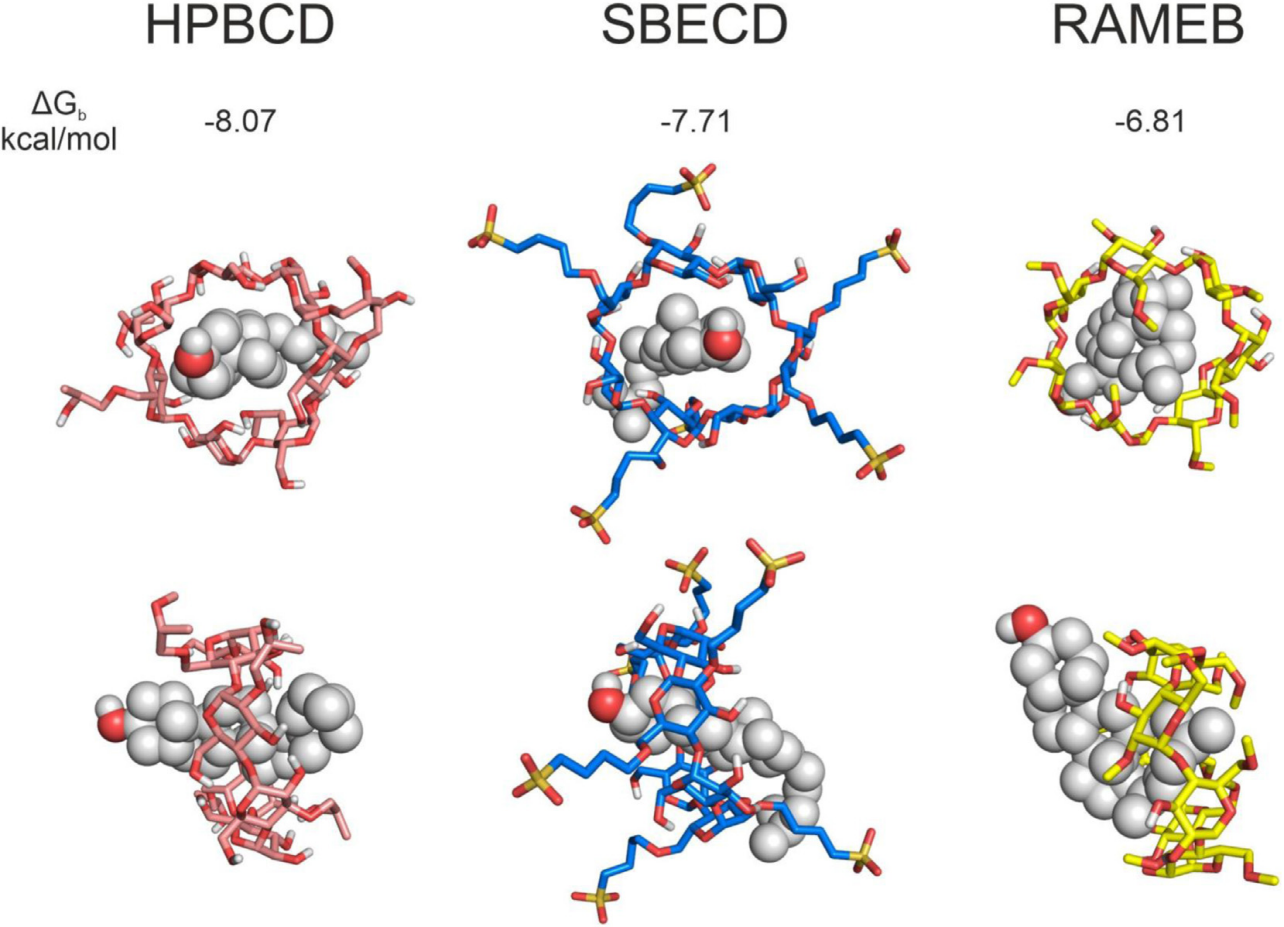
The top and site-view of substituted CD-cholesterol complexes. Cholesterol is shown in *grey*, sphere representation, while 2-hydroxypropyl-beta-cyclodextrin (HPBCD), sulfobutylether-beta-cyclodextrin (SBECD), and randomly methylated beta-cyclodextrin (RAMEB) are in stick representation, coloured by *salmon*, *blue*, and *yellow*, respectively.

The cholesterol-binding mode was similar for HPBCD and SBECD; it docked in the center of the hydrophobic cavity of the hosts. In the case of RAMEB, the steroid part of cholesterol is oriented toward the bulk (Fig. 7). Due to the high DS number, the apolar methyl groups on C6-OH might create hydrophobic interactions with each other in an aqueous environment, resulting in a more closed conformation for the host, which did not allow the ligand to bind deeper. This closed conformation was not characteristic of the hydrophilic substituted CD derivatives, such as HPBCD and SBECD (Fig. 7 bottom). On the other hand, in the case of RAMEB, the abovementioned binding mode of the guest enables connecting an additional host molecule to its steroid part, leading to the development of a more stable 2:1 stoichiometric RAMEB: cholesterol complex. The structural findings were also supported by the energetic data. The slightly more positive ΔG_b_ (Fig. 7) value at RAMEB indicates that the 1:1 stoichiometry is not adequate to achieve a stable complex.

## Discussion

We provide here the first data for the peripheral antinociceptive and anti-vasodilator effects of cholesterol depletion by three CD derivatives, RAMEB, HPBCD and SBECD. Topical pretreatments significantly decreased TRPV1- and TRPA1-mediated acute nocifensive behaviors, mechanical hyperalgesia, and neurogenic vasodilatation. We also demonstrated that local administration of CD derivatives reduced the cholesterol content of the affected area (plantar skin or ear tissue), which supports that their inhibitory actions on nerve terminal activation related to nociception and neurogenic vasodilatation are mediated via cholesterol depletion. The dynamic cholesterol depleting ability of CDs was shown using Filipin III fluorescent labeling and it was demonstrated by *in* silico modeling and docking calculations that the cholesterol binding mode of RAMEB is different from HPBCD and SBECD.

We have earlier demonstrated the receptor inhibiting potential of these CD compounds in vitro in ^45^Ca^2+^-uptake experiment in TRPA1 and TRPV1 receptor-expressing CHO cells (51). The inhibition of TRPV1 and TRPA1 activation via lipid raft disruption has been investigated in various functional experiments and model systems. Even though the dawn of lipid raft research yielded controversial results concerning the relationship of TRP channel activation and lipid microdomains (48, 53, 54, 55), more and more evidence supports the idea that lipid-protein interactions have significant effect on receptor activation. Several research groups revealed that a variety of lipid raft disruptors (MCD, sphingomyelinase (SMase), myriocin, the carboxamido-steroid C1 compound, D-threo-1-Phenyl-2-decanoylamino-3-morpho-lino-1-propanol (D-PDMP), resolvins) altered the membrane composition, resulting in negative functional effects on TRPV1 and TRPA1 receptors in cultured sensory neurons and TRPV1 and TRPA1 receptor-expressing cells (48, 49, 50, 56, 84, 85). Direct evidence has been provided by total internal reflection fluorescent microscopy and gradient density centrifugation that TRPA1 is located in the membrane lipid raft regions. It has been demonstrated that its function is highly dependent on the cholesterol content of the plasma membrane (57, 58).

Although the receptor inhibitory effects of cholesterol depletion have been described from various aspects in several in vitro models, limited knowledge is available about its in vivo consequences on the activation of receptors located on the sensory nerve terminals. There is growing evidence in the literature on different CD-anesthetic combinations, where the CD components increase the analgesic and/or anti-inflammatory effect or have other preferable biopharmaceutical characteristics (stabilization, increased absorption rate, more rapid action, prolonged effect etc.) over the active ingredient alone in various animal models and human studies (86, 87, 88, 89, 90). CDs are also reported to have favorable active pharmaceutical abilities alone in several pathological conditions (e.g lysosomal storage disorders, neurodegenerative disorders) (65, 91, 92). However, their analgesic and anti-inflammatory potentials are highly unexploited. In our recent set of experiments, we showed that the duration of the TRPA1 agonist formalin-induced acute somatic nocifensive behavior was diminished in the second phase of the experiment (20–45 min following formalin administration). The nocifensive behavior in the second phase is mainly arising from the release of inflammatory mediators (CGRP, SP). In the first phase (0–5 min) the direct activation of nerve terminals occurs (70), which was not influenced by the pretreatment. These results support our previous findings, when the cholesterol-depleting MCD and C1 compound exerted similar antinociceptive effect in the same mouse model (61).

AITC, the active component of MO, is known to induce neurogenic inflammation. It has been shown that this inflammation is TRPV1 receptor-independent (76). CD pretreatment reduced MO-induced blood perfusion elevation in mouse ear. As AITC is a selective TRPA1 agonist, these results support the idea that cholesterol depletion leads to decreased activation of raft-associated TRPA1 receptors. Upon TRPA1 activation neuropeptides such as SP and CGRP are released from sensory nerve endings. These neuropeptides contribute to increased vascular permeability and the dilation of blood vessels, which can result in the accumulation of fluid in the tissue, causing oedema (5, 93, 94, 95). It has been previously described that neurokinin 1 receptor (NK1R) activated by SP also plays a pivotal role in MO-induced ear swelling (76, 96, 97, 98), and it is also known that NK1Rs reside in the lipid rafts of the plasma membrane (99, 100). Although a tendency was observed that ear thickening was less pronounced following 30-min CD pretreatment, this difference in the oedema size was not significant in the first 4 h of the experiment.

I.pl. administration of the ultrapotent TRPV1 agonist RTX induces mechanical hyperalgesia predominantly via peripheral sensitization and thermal allodynia involving central sensitization mechanisms (72). Peripheral RAMEB, HPBCD, and SBECD pretreatment significantly alleviated RTX-evoked mechanical hyperalgesia. Remarkable alteration in the thermal nociception of the animals was not observed. These results are in accordance with our previous findings, where we demonstrated similar pattern with TRPV1 inhibition by the lipid raft disruptor SMase and C1 compound (61, 62). The fact that the local administration of the compounds did not affect the thermal perception may be advantageous, regarding that due to their thermosensitive nature targeting TRPV1 and TRPA1 is highly challenging, as the loss of thermonociception results in undesirable events (e.g burning accidents) (101). RTX-induced neuropathic pain was alleviated by MCD treatment through phosphatidylinositol 4,5-bisphosphate hydrolysis in mice (63). In another study it was shown that local and systemic RAMEB treatment reduced the Complete Freund’s adjuvant-induced thermal and mechanical hyperalgesia in rats (102).

To further support our in vivo findings, we showed the cholesterol-depleting effect of the CD derivatives by measuring the cholesterol content of the plantar skin or ear tissue of mice in cases of i.pl. injection or smearing the CD derivatives on the surface of the ear. This in vitro evidence of cholesterol depletion supports our hypothesis that the antinociceptive and anti-inflammatory effects are arising from the disruption of the lipid rafts, that plays a crucial role in TRPV1 and TRPA1 functioning. The cholesterol depleting effect of CDs has been also confirmed in several in vitro studies.

Cholesterol depletion of CD treatment was also investigated in vitro on CHO cells by the selective cholesterol-binding Filipin III fluorescent dye by confocal microscopy. Our research group already demonstrated with this method that different CDs as well as C1 compound depleted cholesterol from the plasma membrane of CHO cells (48, 50, 51). In our recent study we investigated the dynamic nature of cholesterol depletion of RAMEB, HPBCD and SBECD with different approaches. In our control group the applied CDs were shown to reduce cholesterol content of CHO cells, which is in accordance with previous results (51). In the first, restorative approach CHO cells’ cholesterol content was restored by CLC treatment following depletion by CDs. It has been previously proven that this method can replete cell cholesterol levels (103, 104). After CD treatment restoration resulted in recovery of the cells’ cholesterol levels to the untreated control level, suggesting the reversibility of the cholesterol depletion after treatment. This is an important aspect regarding the possible application of CD derivatives as analgesic compounds. However, cholesterol overloading prior to CD treatment did not prevent cholesterol depletion of the derivatives, supporting the significant cholesterol-depleting effect of CD derivatives. These approaches together strengthen the idea that CDs can be applied as potent, but reversible agents in cholesterol depletion.

Our in silico investigations revealed some differences between the cholesterol-binding mode of the methylated derivative RAMEB compared to the non-methylated derivatives HPBCD and SBECD, which showed higher similarity to each other. Docking cholesterol to the hydrophilic substituted HPBCD and SBECD resulted in cholesterol binding to the center of the host’s cavity. RAMEB, due to its numerous apolar methyl groups on C6-OH achieved a more closed conformation and less deep binding of the guest molecule. This conformation may also promote the binding of an additional host molecule to cholesterol’s steroid part, leading to the development of a more stable 2:1 stoichiometric RAMEB:cholesterol complex. Literature data support these findings: at low host concentration, the 1:1 stoichiometric HPBCD-cholesterol complex is relatively stable (105), while RAMEB prefers a 2:1 stoichiometry (106, 107). The structural findings were also supported by the energetic data. The slightly more positive ΔG_b_ value at RAMEB indicates that the 1:1 stoichiometry is not adequate to achieve a stable complex. This finding agrees with previous studies performed for unmodified CDs (106), that also concluded a 2:1 stoichiometry necessary for stable complex formation. These results also show an interesting perspective, proposing a potential explanation of the better cholesterol-depleting ability of methylated CD derivatives, like RAMEB, and consequently their stronger cytotoxicity even in lower concentrations.

All three investigated derivatives exerted similar significant effects in our applied acute pain and neurogenic vasodilation models. The methylated derivative RAMEB was more potent than the non-methylated derivatives HPBCD and SBECD, as it gave the same favorable results in a lower concentration. However, it is also important to take into consideration that this lower concentration may not be safely exceeded due to toxicity issues, potentially relating to the methylated nature of the derivative, supported by in vitro and in silico findings. We conclude that local treatments with these safer, modified CDs may be powerful therapeutic tools for peripheral analgesic and anti-inflammatory actions presumably via cholesterol depletion and possibly via lipid raft disruption.

## Data availability

Data are to be shared upon request. Please contact Dr Éva Szőke (eva.szoke@aok.pte.hu).

## Supplemental data

This article contains supplemental data.

## Conflict of interest

The authors declare that they have no conflicts of interest with the contents of this article.

## Supplemental Data

Supplementary figure Flowchart of the acute pain models and *in vitro* investigations: (A) Formalin-induced acute somatic behavior test, (B) RTX-induced acute thermal allodynia and mechanical hyperalgesia model, (C) Mustard oil-induced acute skin inflammation model in mouse ear, (**D**) Colorimetric cholesterol content measurement. Created in BioRender. Bencze, N. (2025) https://BioRender.com/ke4njr0.
