# Supplementary figures and images for: Cyclodextrins inhibit TRPV1 and TRPA1 activation-induced nociception via cholesterol depletion
Source: J Lipid Res. 2025 Jun 16;66(7):100844. doi: 10.1016/j.jlr.2025.100844 (PMC12274755; doi:10.1016/j.jlr.2025.100844)

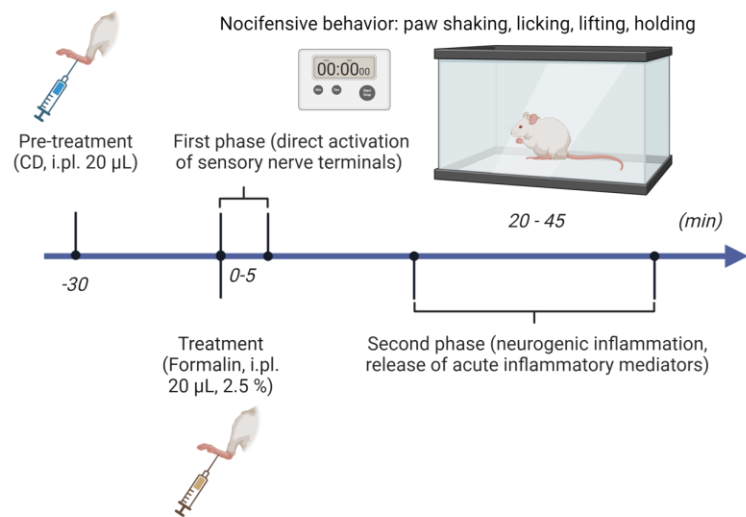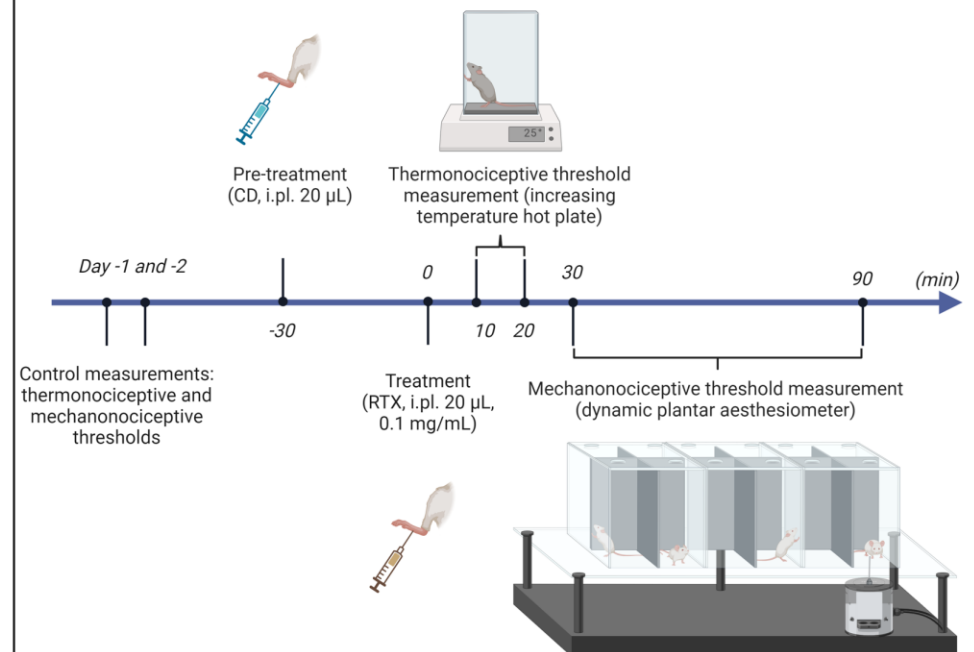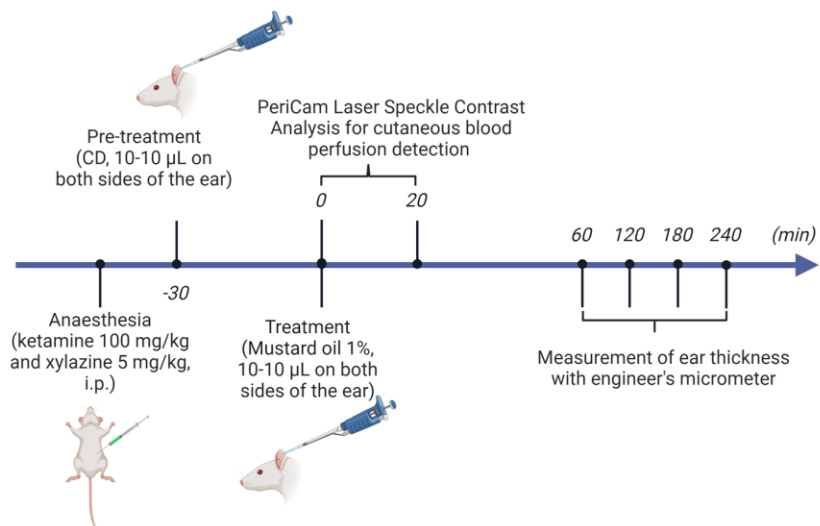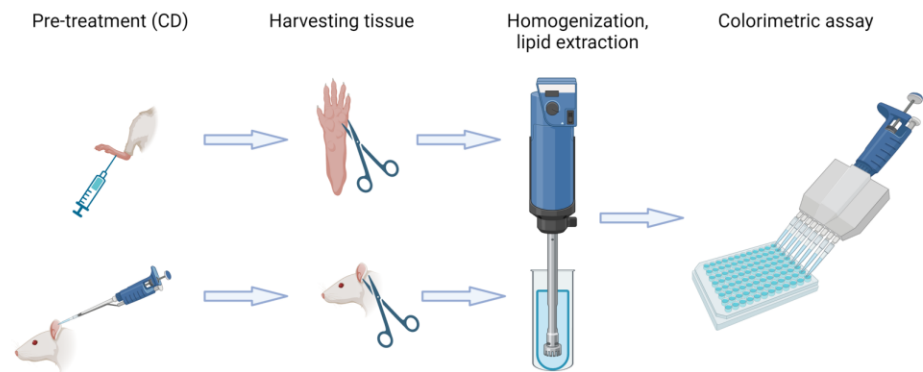

Supplement: Supplementary figure — Flowchart of the acute pain models and in vitro investigations: (A) Formalin-induced acute somatic behavior test, (B) RTX-induced acute thermal allodynia and mechanical hyperalgesia model, (C) Mustard oil-induced acute skin inflammation model in mouse ear, (D) Colorimetric cholesterol content measurement. Created in BioRender. Bencze, N. (2025) https://BioRender.com/ke4njr0. [file mmc1.pdf]
